# Septin 9 PB domains coordinate centrosome positioning and microtubule acetylation to control epithelial polarity

**DOI:** 10.1002/1873-3468.70363

**Published:** 2026-05-26

**Authors:** Ting ting Cai, Mohyeddine Omrane, Nassima Benzoubir, Didier Samuel, Ama Gassama‐Diagne

**Affiliations:** ^1^ INSERM, Unité 1193 Villejuif France; ^2^ Université Paris Saclay, UMR‐S 1193 Villejuif France; ^3^ AP‐HP Hôpital Paul Brousse, Centre Hepato‐Biliaire Villejuif France

**Keywords:** centrosome positioning, epithelial polarity, HDAC6, microtubule acetylation, septins, TGF‐β signaling

## Abstract

Septins are cytoskeletal GTP‐binding proteins that organize microtubules and scaffold polarity complexes. Here, we uncover a polybasic (PB)‐domain‐dependent mechanism by which septin 9 controls apico‐basal polarity in epithelial cells. Septin 9 regulates centrosome positioning and the asymmetric distribution of acetylated microtubules, which are required for ciliogenesis and lumen formation. Knockdown of septin 9 or deletion of its PB domains disrupts centrosome asymmetry, induces a symmetric distribution of acetylated tubulin, and impairs cilia formation, leading to polarity inversion. These defects are associated with increased expression of the microtubule deacetylase HDAC6. Importantly, inhibition of TGF‐β signaling or selective HDAC6 inhibition with tubacin restores microtubule acetylation and rescues apico‐basal polarity. Together, our findings identify septin 9 as a cytoskeletal integrator that links centrosome positioning and microtubule acetylation to epithelial morphogenesis through the TGF‐β/HDAC6 pathway.

## Graphical abstract

Septin 9 polybasic domains couple phosphoinositide‐rich membrane binding to centrosome positioning, Golgi organization, and microtubule acetylation to control epithelial polarity. Their loss disrupts this axis, causing centrosome mispositioning, Golgi fragmentation, reduced microtubule acetylation, and polarity inversion via upregulation of the microtubule deacetylase HDAC6.

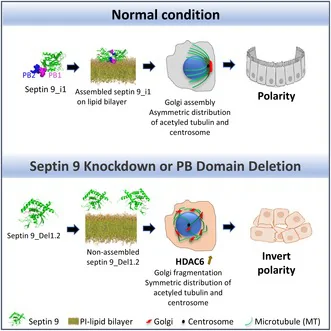

## Abbreviations

**2D, 3D**, two and three dimensional

**ECM**, extracellular matrix

**EMT**, epithelial–mesenchymal transition

**EV**, empty vector

**FIJI**, image processing software

**HDAC6**, histone deacetylase 6

**Icy**, bioimage analysis software

**MDCK**, Madin–Darby canine kidney

**MT**, microtubule

**MTOC**, microtubule‐organizing center

**PB1, PB2**, polybasic domain 1 and 2

**PIs**, phosphoinositides

**ROI**, region of interest

**TGF‐β**, transforming growth factor beta

Epithelial cells exhibit apico‐basal polarity, characterized by an apical surface facing the lumen and a basolateral domain contacting neighboring cells and the extracellular matrix (ECM). This polarity arises from the asymmetric organization of membranes, cytoskeletal elements, and intracellular organelles and is essential for epithelial tissue function [1]. Disruption of apico‐basal polarity has profound pathological consequences, particularly in carcinoma, underscoring the importance of elucidating the molecular mechanisms that establish and maintain epithelial organization [2].

The establishment of apico‐basal polarity is a multistep process initiated by cues from the ECM and cell–cell contacts, which trigger cytoskeletal reorganization, organelle repositioning, and polarized trafficking of membrane components. These coordinated events define the apico‐basal axis and enable lumen formation [3, 4]. During this process, primary cilia assemble at the apical membrane and protrude into the lumen, where they function as signaling hubs essential for epithelial differentiation and tissue homeostasis. Ciliogenesis is therefore tightly coupled to polarity regulators and cytoskeletal organization [5, 6, 7].

The Golgi–centrosome–microtubule network plays a central role in epithelial polarity by directing polarized membrane trafficking and coordinating organelle positioning. The Golgi apparatus acts as a key organizer of vesicular transport [8, 9], and its positioning is a critical determinant of trafficking directionality and the spatial distribution of polarity cues [10]. The Golgi and centrosome function as a coordinated unit during cell polarization [11] connected through microtubules that support directional transport and cell morphogenesis [12]. Early studies in MDCK epithelial cells demonstrated that polarization is accompanied by reorganization of microtubule‐nucleation sites and a marked increase in microtubule stability [13]. Conversely, disruption of the microtubule network leads to Golgi fragmentation and dispersion [14, 15] and correlates with reduced microtubule acetylation [16], underscoring the importance of microtubule stability in maintaining Golgi organization and polarity. More recent work has revealed additional complexity in microtubule organization during epithelial morphogenesis. In developing epithelia, distinct microtubule‐organizing centers (MTOCs), including centrosomal and noncentrosomal MTOCs, generate microtubule populations with different dynamics and nucleation requirements [17]. These observations suggest that, beyond classical centrosome‐based nucleation, specialized cytoskeletal scaffolds may spatially specify stabilized microtubule arrays required for epithelial architecture. However, the molecular mechanisms that coordinate centrosome positioning with the asymmetric stabilization of microtubules during epithelial polarization remain incompletely understood.

Membrane identity and curvature, governed in part by phosphoinositides (PIs), are critical for cytoskeletal organization and epithelial morphogenesis. Although they represent a minor fraction of total cellular lipids, PIs play pivotal roles in signaling, vesicular trafficking, and cytoskeletal regulation [18]. By recruiting specific protein partners to defined membrane domains, PIs orchestrate cortical organization and signaling cascades that control polarity establishment and lumen formation [19, 20].

Septins are GTP‐binding cytoskeletal proteins that assemble into hetero‐oligomeric complexes and higher‐order structures, such as filaments and rings, which scaffold and organize actin and microtubule networks [21]. Thirteen septin family members and multiple isoforms contribute to the structural and functional diversity of these assemblies. Septins associate with membranes through binding to PIs via a well‐characterized polybasic domain (PB1) [22, 23, 24], as well as a second polybasic domain (PB2) located within the GTP‐binding region and conserved across the septin family [25]. We previously demonstrated that both PB domains are essential for septin 9 filament assembly and Golgi organization [25] and that deletion of these domains disrupts apico‐basal polarity in three‐dimensional (3D) epithelial cultures [26]. However, whether septin 9 and specifically its PB domains coordinate centrosome positioning with the asymmetric stabilization of microtubules during epithelial morphogenesis has remained unclear.

Here, we demonstrate that septin 9 PB domains are critical for centrosome positioning, asymmetric distribution of acetylated microtubules, and ciliogenesis in both two‐dimensional and three‐dimensional (3D) epithelial models. We show that knockdown of septin 9 or deletion of its PB domains disrupts microtubule acetylation and its spatial organization within the cytoplasm and primary cilium. These defects are associated with elevated expression of the microtubule deacetylase HDAC6 and microtubule destabilization in cysts exhibiting inverted polarity. Importantly, pharmacological inhibition of HDAC6 with Tubacin or blockade of TGF‐β signaling restores acetylated microtubule organization and rescues epithelial polarity. Together, our findings identify septin 9 as a cytoskeletal integrator that links centrosome positioning and microtubule acetylation to epithelial morphogenesis through the TGF‐β/HDAC6 signaling axis.

## Materials and methods

### Cell lines and culture conditions

HeLa (CVCL_0030) SilenciX Septin 9 knockdown and control cell lines were from Tebu‐bio, Le Perray‐en‐Yvelines, France. Cells were maintained in Dulbecco's modified Eagle's medium (DMEM; Invitrogen, Illkirch‐Graffenstaden, France) containing 4.5 g·L^−1^ glucose and supplemented with 10% heat‐inactivated fetal bovine serum (FBS), 1% nonessential amino acids (Gibco BRL, Illkirch‐Graffenstaden, France), and 1% penicillin/streptomycin (10 000 U·mL^−1^ and 10 000 μg·mL^−1^; Gibco BRL), together with hygromycin B (100 μg·mL^−1^; Invitrogen). MDCK (Madin–Darby Canine Kidney) (CVCL_0422) cells, from Keith Mostov (UCSF, San Francisco, CA, USA), were cultured in minimal essential medium (MEM; Invitrogen) containing Earle's balanced salt solution, supplemented with 5% FBS and 1% penicillin/streptomycin, at 37 °C in 5% CO₂.

For 2D culture, cells were seeded on coverslips in 12‐well plates and grown for 48 h. For 3D culture, cells were trypsinized, resuspended at 10 000 cells·mL^−1^ in 2% Matrigel (BD Biosciences, Noyal‐Châtillon‐sur‐Seiche, France), and 500 μL of cell suspension was plated into 8‐well Lab‐Tek II chamber slides (Thermo Fisher Scientific, Illkirch‐Graffenstaden, France) precoated with Matrigel. Cysts were allowed to develop for 4 days until lumen formation, as described previously [27].

All cell lines were authenticated using complementary approaches. MDCK cells were verified by species‐specific PCR using canine‐specific primers and display a characteristic 3D cyst‐forming morphology, providing further confirmation of their identity. HeLa Septin 9 knockout (KO) cell lines were routinely validated by immunoblotting to confirm the absence of the target protein. All experiments were performed using mycoplasma‐free cells, and mycoplasma contamination was routinely monitored on a monthly basis using a Mycoplasma Detection Kit (Lonza, Basel, Switzerland).

### Plasmid and siRNA

pcDNA constructs encoding Septin 9 isoform 1 (Septin 9_i1), isoform 4 (Septin 9_i4), and the empty vector (EV) were provided by Dr. Russell (CCRCB, Queen's University Belfast).

cDNAs encoding Septin 9_i1, Septin 9_i4, the PB1‐deleted mutant (Septin 9_del1), and the PB1/PB2 double‐deleted mutant (Septin 9_del1.2) in the pcDNA3.1/V5‐His‐TOPO backbone have been described previously [25, 26].

Septin 9 duplex siRNAs (Thermo Fisher), and scrambled control siRNA (Santa Cruz, Heidelberg, Germany) were used.

Sequences:

- si SEPT9 (HSS173896, si2): 5′‐AGGCGCCUGCAUCACGGAACGAGAA‐3′; 5′‐UUCUCGUUCCGUGAUGCAGGCGCCU‐3′;
- si SEPT9 (HSS173897, si3): 5′‐GCCAUGAAGCAGGGCUUCGAGUUCA‐3′; 5′‐UGAACUCGAAGCCCUGCUUCAUGGC‐3′.

### Drug treatments

MDCK cells were treated with 33 nm nocodazole (Calbiochem, Fontenay‐sous‐Bois, France; Cat#487928) for 24 h in 2D culture. For 3D cultures, MDCK cysts were treated with SB431542 at 2 μm (MedChemExpress, Nanterre, France; Cat#HY‐10431) for 5 days, with daily medium replacement. Tubacin at 1 μm (MedChemExpress, Cat#HY‐13428) was administered for 5 days, with daily medium and drug renewal.

### Antibodies

Primary antibodies:

- Anti‐Septin 9 (Abcam, Amsterdam, Netherlands; Cat#ab38314; WB 1 : 500, IF 1 : 25)
- Anti‐α‐tubulin (Abcam, Cat#ab15246; WB 1 : 2000, IF 1 : 100)
- Anti‐giantin (Abcam, Cat#ab37266; IF 1 : 100)
- Mouse and rabbit anti‐V5 (Abcam, Cat#ab27671 and Cat#ab9116; WB 1 : 1000, IF 1 : 400)
- Anti‐actin (Santa Cruz, Cat#sc‐1616; WB 1 : 1000)
- Anti‐GM130 (BD Transduction Laboratories, Noyal‐Châtillon‐sur‐Seiche, France; Cat#610822; IF 1 : 50)
- Anti‐TGN46 (Novus, Lille, France; Cat#NB110‐62093; IF 1 : 50)
- Anti‐HDAC6 (Santa Cruz, Cat#sc‐11 420; WB 1 : 1000, IF 1 : 50)
- Anti‐HEF1 (Abcam, Cat#ab18056; WB 1 : 500)

Secondary antibodies conjugated to Alexa Fluor^®^ 488, 546, or 633 were purchased from Life Technologies, Illkirch‐Graffenstaden, France. Phalloidin Labeling Probes Alexa Fluor Plus 647 (Invitrogen, Cat#A30107, IF 1/1000). Hoechst 33342 was obtained from Molecular Probes.

### Immunofluorescence staining

Cells grown on coverslips were fixed in 4% paraformaldehyde for 15 min at 4 °C and permeabilized in PFS buffer (PBS containing 0.025% saponin and 0.7% gelatin) for 30 min at 37 °C. Samples were incubated with primary antibodies for 2 h at room temperature, followed by secondary antibodies for 90 min. Nuclei were stained with Hoechst 33342 (Fisher Scientific, Cat#15831459). Coverslips were mounted with ProLong Gold (Invitrogen). For 3D cultures, samples were incubated in primary antibodies overnight at 4 °C, followed by secondary antibody incubation for 90 min at 37 °C, then processed as above.

### Image acquisition and analysis

Images were acquired using a Leica TCS SP5 AOBS tandem confocal microscope. 3D reconstructions were generated using Icy Bioimage Analysis Software (icy 2.5.4.0; Icy (Institut Pasteur), Paris, France).

For intensity quantification of intracellular compartments, confocal Z‐stacks were background‐subtracted in imagej 1.54, ImageJ (National Institutes of Health), Bethesda, MD, USA. Cell ROIs were defined using the Free Selection tool, intensities outside ROIs were cleared, and normalized intensity values along concentric regions were measured using the Radial Profile Angle plug‐in. The nuclear center was automatically determined from the nuclear ROI.

### Immunoblotting

Cells were lysed in Duchenne phosphate buffer (DPBS) containing 20 mm Tris/HCl, 100 mm NaCl, 1% Triton X‐100, 10 mm EDTA, and protease/phosphatase inhibitors (Roche, Meylan, France). Proteins were separated by SDS/PAGE and transferred to nitrocellulose membranes. After blocking, membranes were incubated with primary and HRP‐conjugated secondary antibodies and developed using ECL (Thermo Scientific). Signals were acquired using ECL Plus (Thermo Scientific, Cat#32132) and imaged with a G: BOX Chemi System (Syngene, Ozyme, Saint‐Cyr‐l'École, France). Band intensities were quantified using imagej.

### qRT‐PCR

Total RNA was extracted with the RNeasy Mini Kit (Qiagen, Courtaboeuf, France) and reverse transcribed using the RevertAid cDNA Synthesis Kit (Thermo Scientific cat# K1622). qPCR was performed with Roche‐LightCycler^®^ 480 SYBR Green I Master (cat# 4707516001) on a LightCycler^®^ 96 Instrument (Roche). Gene expression was normalized to GAPDH. Primer sequences are listed in Table S1.

### Statistical analysis

Data from immunofluorescence, immunoblotting, and qRT‐PCR were analyzed using two‐tailed Student's *t*‐tests (Excel 2019, prisme 8.4.3; GraphPad Prism 8.4.3 (GraphPad Software), San Diego, CA, USA). Data are presented as mean ± SEM (standard error of the mean). Differences between groups were analyzed using Student's *t*‐test. A *P*‐value <0.05 was considered statistically significant.

## Results

### Septin 9 regulates nucleus‐centrosome positioning

The centrosome serves as the main microtubule‐organizing center (MTOC) in animal cells, promoting *de novo* microtubule (MT) assembly and organizing the Golgi apparatus [28, 29]. In mammalian cells, the Golgi is organized into stacks of flattened cisternae laterally connected to form a ribbon near the centrosome, critical for polarized trafficking and apico‐basal organization [11].

We previously reported that septin 9 regulates MT‐dependent Golgi assembly [25] and controls apico‐basal polarity [26]. To determine whether septin 9 also regulates centrosome positioning, HeLa cells were transfected with a V5‐tagged isoform 1 of septin 9 (septin 9_i1), and stained for the V5 tag, γ‐tubulin (centrosome marker), and the trans‐Golgi network marker TGN46. Immunofluorescence analysis suggested that Golgi structures are in close proximity to septin 9 filaments that appear to converge toward the centrosomal region (Fig. 1A). To assess whether septin 9 depletion affects centrosome localization, cells were transfected with septin 9 siRNA and stained for γ‐tubulin and Giantin or TGN46 (Golgi marker) (Fig. 1B, Fig. S1). In control cells, centrosomes localized adjacent to the nucleus and remained closely associated with the Golgi, as expected. In contrast, septin 9‐depleted cells displayed striking defects in centrosome positioning; in approximately 60% of cells, centrosomes were displaced to a central position above the nucleus (Fig. 1C). Immunoblotting confirmed efficient septin 9 knockdown (Fig. 1D). Together, these data demonstrate that septin 9 is required for proper centrosome positioning relative to the nucleus.

**Fig. 1:**
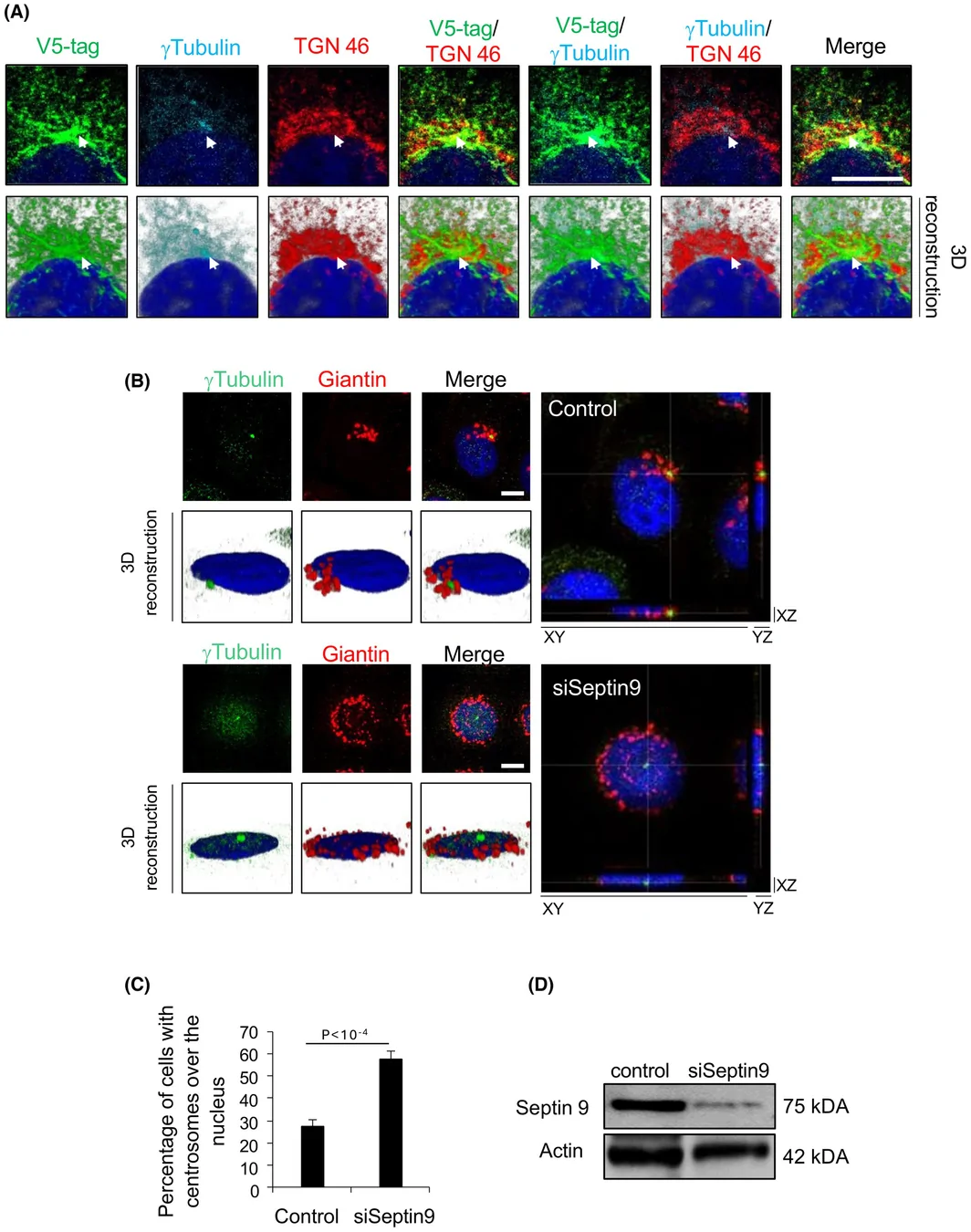
Septin 9 localizes to centrosomes and controls their positioning. (A) HeLa cells expressing V5‐septin 9_i1 were stained for V5 tag (green), TGN46 (red) as a Golgi marker, γ‐tubulin (blue), and nuclei (blue). Representative 2D/3D images are shown; arrowheads indicate centrosomes. Scale bar: 5 μm. (B) Control and septin 9‐depleted using siRNA (siSeptin9) cells were stained for γ‐tubulin (green), Giantin (red) as a Golgi marker, and nuclei (blue). Representative 2D/3D images and XZ projections are shown. Scale bar: 10 μm. Graph shows the percentage of cells (*n* = 700) with centrosomes positioned above the nucleus (mean ± SEM, two experiments). (C) Quantification of centrosome positioning. Data are shown as mean ± SEM. Statistical analysis was performed using Student's *t*‐test (*P* < 0.05). (D) Immunoblot verifying septin 9 depletion; actin was used as loading control.

### Septin 9 promotes asymmetric distribution of acetylated microtubules

Microtubule acetylation stabilizes MT filaments and contributes to Golgi anchoring [11, 28]. In HeLa cells expressing septin 9_i1, acetylated α‐tubulin filaments converged asymmetrically on one side of the nucleus and colocalized with Golgi‐associated septin 9 filaments (Fig. 2A). In contrast, septin 9 depletion resulted in a symmetric distribution of acetylated microtubules around the nucleus, concomitant with Golgi fragmentation (Fig. 2B).

**Fig. 2:**
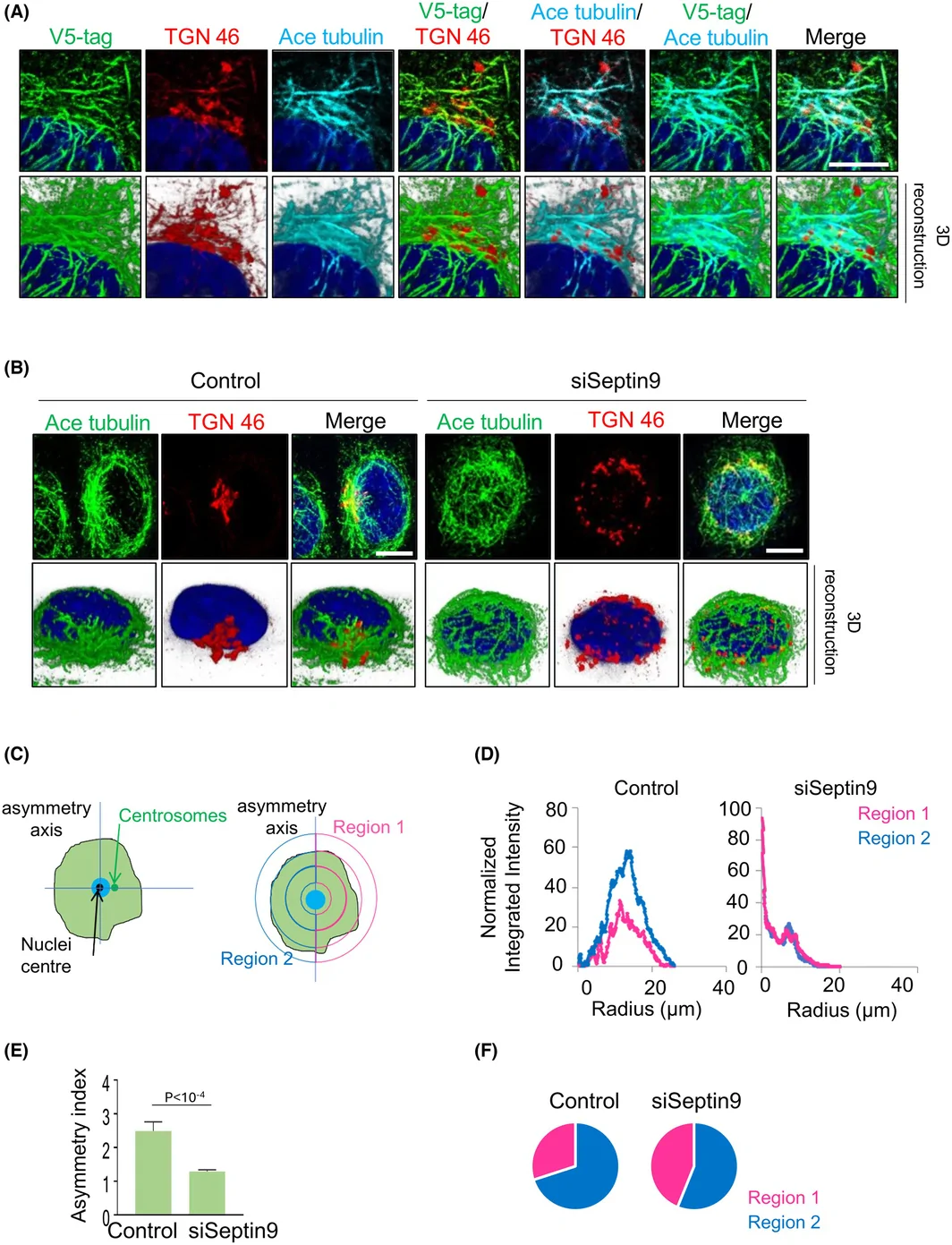
Septin 9 colocalizes with acetylated microtubules and maintains their asymmetry. (A) HeLa cells expressing septin 9_i1 were stained for V5‐tag (green), TGN46 (red) as a Golgi marker, acetylated α‐tubulin (Ace‐tubulin, blue), and nuclei (blue). Representative 2D/3D images. Scale bar: 5 μm. (B) Control cells and cells which septin 9‐silenced using siRNA (siSeptin9) cells stained for acetylated α‐tubulin (green), TGN46 (red), and nuclei. Scale bar: 10 μm. (C) Schematic of the asymmetry axis and the regions used for intensity analysis. (D) Radial intensity profiles of acetylated α‐tubulin in each region. Integrated intensities were normalized between regions 1 and 2. Fluorescence intensities were measured using the Radial Profile Angle plug‐in in FIJI and corrected for background. (E) Difference in intensity between region 2 and region 1, used as an asymmetry index to quantify the distribution of acetylated α‐tubulin between the two regions. Data represent *n* = 23 control cells and *n* = 33 septin 9_del1.2 cells. Data are shown as mean ± SEM. Statistical analysis was performed using Student's *t*‐test (*P* < 0.05). (F) Percentage distribution of acetylated α‐tubulin intensity between regions A and B, representing the fraction of total cellular fluorescence. Sector plots illustrate the asymmetric distribution of acetylated α‐tubulin.

To quantitatively assess this effect, each cell was divided into two symmetric regions along the nuclear axis (regions 1 and 2). Radial profile analysis revealed a significant loss of asymmetry in acetylated microtubule distribution upon septin 9 depletion (Fig. 2C–F). These data demonstrate that septin 9 is required for the asymmetric organization of acetylated microtubules.

### Septin 9 PB domains regulate γ‐tubulin recruitment and centrosome positioning by stabilizing microtubules

To dissect the mechanistic role of septin 9 polybasic (PB) domains in centrosome positioning and microtubule organization, polarized MDCK cells were stably transfected to express an empty vector (septin 9_EV), full‐length septin 9 isoform 1 (septin 9_i1), or a PB‐domain‐deleted mutant (septin 9_del1.2).

In polarized epithelial cells, the centrosome and Golgi are maintained in an asymmetric apical position through tight coupling between the microtubule cytoskeleton and membrane‐associated polarity cues [30]. Consistent with this organization, septin 9_EV and septin 9_i1 expressing cells displayed asymmetric centrosome and Golgi positioning, with septin 9_i1 further enhancing Golgi compaction (Fig. 3A–C). Proper expression of each construct was confirmed by immunoblotting (Fig. 3D). These observations suggest that septin 9 actively promotes stabilization and spatial confinement of the apical microtubule network.

**Fig. 3:**
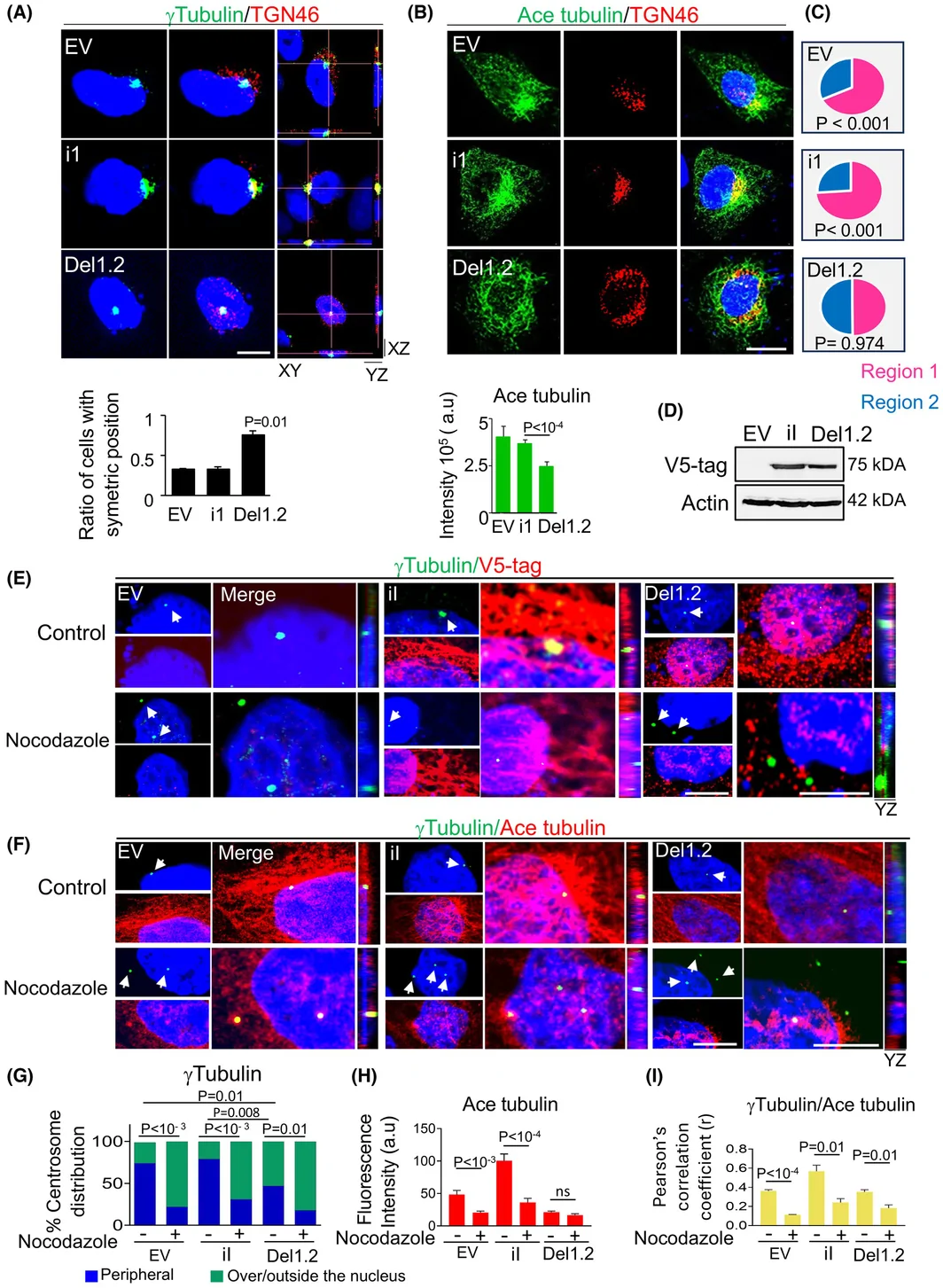
PB‐domain deletion in septin 9 disrupts centrosome and microtubule asymmetry. (A) Cells stained for γ‐tubulin (green), TGN46 as a Golgi marker (red), and nuclei (blue). Representative images and XZ views illustrate centrosome position. Scale bar: 10 μm. Adjacent quantification (*n* = 851) shows reduced centrosome asymmetry (*P* < 0.001, *P* < 0.01). (B) Cells were stained for acetylated α‐tubulin (Ace‐tubulin, green), TGN46 (red) as a Golgi marker, and nuclei (blue). Total fluorescence intensity was quantified (*n* = 35). Data are shown as mean ± SEM. Statistical analysis was performed using Student's *t*‐test (*P* < 0.05). (C) Sector analysis of acetylated α‐tubulin distribution (*n* = 15). (D) Immunoblot analysis of V5‐Septin 9‐I1, septin 9_del1.2 expression in stable MDCK cell lines. Actin was used as a control loading. (E) MDCK stable cell lines expressing empty vector (EV), Septin 9 (i1), or septin 9_del1.2 were cultured under 2D conditions and treated or not with 33 nm nocodazole for 2 h. Cells were immunostained for γ‐tubulin (green), V5‐tag (red), and nuclei (blue). A single confocal section and XZ views of centrosome positioning are presented. Colocalization coefficients were calculated for the indicated stains. White arrowheads indicate centrosomes. Scale bar, 10 μm. (F) Cells under the same conditions were stained for γ‐tubulin (green), acetylated α‐tubulin (red), and nuclei (blue) XZ views showing centrosome positioning are presented. White arrowheads indicate centrosomes Scale bar: 10 μm. (G) Quantification of centrosome distribution patterns. Data are expressed as percentages from 50 cells per condition in independent experiments (*n* = 3). Data are shown as mean ± SEM. Statistical analysis was performed using Student's *t*‐test (*P* < 0.05). (H) Quantification of acetylated α‐tubulin fluorescence intensity under the indicated conditions Data are shown as mean ± SEM. Statistical analysis was performed using Student's *t*‐test (*P* < 0.05). n.s. indicates not statistically significant. (I) Colocalization coefficients were calculated under the indicated conditions using Pearson's correlation coefficient (*n* = 9) Data are shown as mean ± SEM. Statistical analysis was performed using Student's *t*‐test (*P* < 0.05).

Strikingly, deletion of the PB domains disrupted this organization; in septin 9_del1.2 cells, the centrosome relocated to a central position above the nucleus, phenocopying septin 9 depletion (Fig. 1). This finding indicates that the PB domains are essential for anchoring or retaining the centrosome within a polarized cortical microtubule architecture.

In parallel, acetylated microtubules, marking long‐lived, stabilized microtubule populations, lost their polarized distribution and instead formed a symmetric perinuclear network associated with fragmented Golgi stacks (Fig. 3A–C). The concomitant reduction in total acetylated tubulin levels (Fig. 3B) suggests that loss of PB domains compromises microtubule stabilization rather than simply redistributing pre‐existing microtubules. Together, these findings support a model in which septin 9, via its PB domains, stabilizes and spatially confines acetylated microtubules to maintain centrosome asymmetry and Golgi integrity in polarized cells.

### Centrosome positioning by septin 9 depends on stabilized acetylated microtubules

To directly examine the contribution of microtubules to septin 9‐dependent centrosome positioning, septin 9_EV, septin 9_i1, and septin 9_del1.2 cells were treated or not with nocodazole and stained for β‐tubulin and septin 9 (V5 tag) (Fig. S2A). As expected, nocodazole treatment disrupted both septin 9_i1 filaments and the microtubule network; however, the disassembled structures remained colocalized under all conditions (Fig. S2B).

We next analyzed centrosome organization by staining for septin 9 and γ‐tubulin (Fig. 3E). In septin 9_i1 cells, nocodazole treatment dispersed γ‐tubulin into multiple foci and abolished its asymmetric localization. In septin 9_del1.2 cells, γ‐tubulin, initially positioned above the nucleus, was redistributed into numerous foci distant from the nucleus following nocodazole treatment (Fig. 3F,G).

Staining for γ‐tubulin and acetylated tubulin revealed that nocodazole treatment reduced acetylated tubulin levels in both septin 9_i1 and septin 9_del1.2 cells (Fig. 3F,H). Moreover, nocodazole significantly decreased the colocalization between γ‐tubulin and acetylated tubulin (Fig. 3F,I). These results indicate that septin 9‐mediated centrosome recruitment and positioning depend on the presence of stabilized, acetylated microtubules.

### PB domains of septin‐9 are required for apical microtubule acetylation and ciliogenesis

In polarized epithelia, acetylated microtubules accumulate at the apical side [31] and form the core of the primary cilium protruding into the lumen [6, 32]. To determine whether deletion of the PB domains affects ciliogenesis, MDCK cysts were analyzed in 3D culture. As previously reported, EV‐ and septin 9_i1‐expressing cells formed cysts with a single open lumen, with β‐catenin marking the apico‐basal junctions. In these cysts, septin 9_i1 localized basolaterally. In contrast, expression of the septin 9_del1,2 mutant induced either multilumen cysts or polarity inversion, and the mutant protein displayed a diffuse cytoplasmic distribution (Fig. 4A) [26]. Because septin 9 assembly into septin 2/6/7 octamers is required for its function, septin 7 expression was also examined. Septin 7 localized correctly to the basolateral membrane, and septin 9 depletion reduced septin 7 levels, consistent with previous findings [26] (Fig. S3). Under these 3D conditions, control and septin 9_i1 cysts displayed open lumens enriched in apical acetylated tubulin and well‐defined luminal cilia. Septin 9_i1 expression further increased acetylated tubulin levels and promoted the formation of elongated cilia (Fig. 4B). In contrast, inverted polarity septin 9_del1,2 cysts exhibited peripheral accumulation of acetylated tubulin and mislocalized cilia (Fig. 4B). Together, these results demonstrate that the PB domains of septin 9 are essential for centrosome positioning, apical microtubule acetylation, and ciliogenesis, key processes underlying proper apico‐basal polarity.

**Fig. 4:**
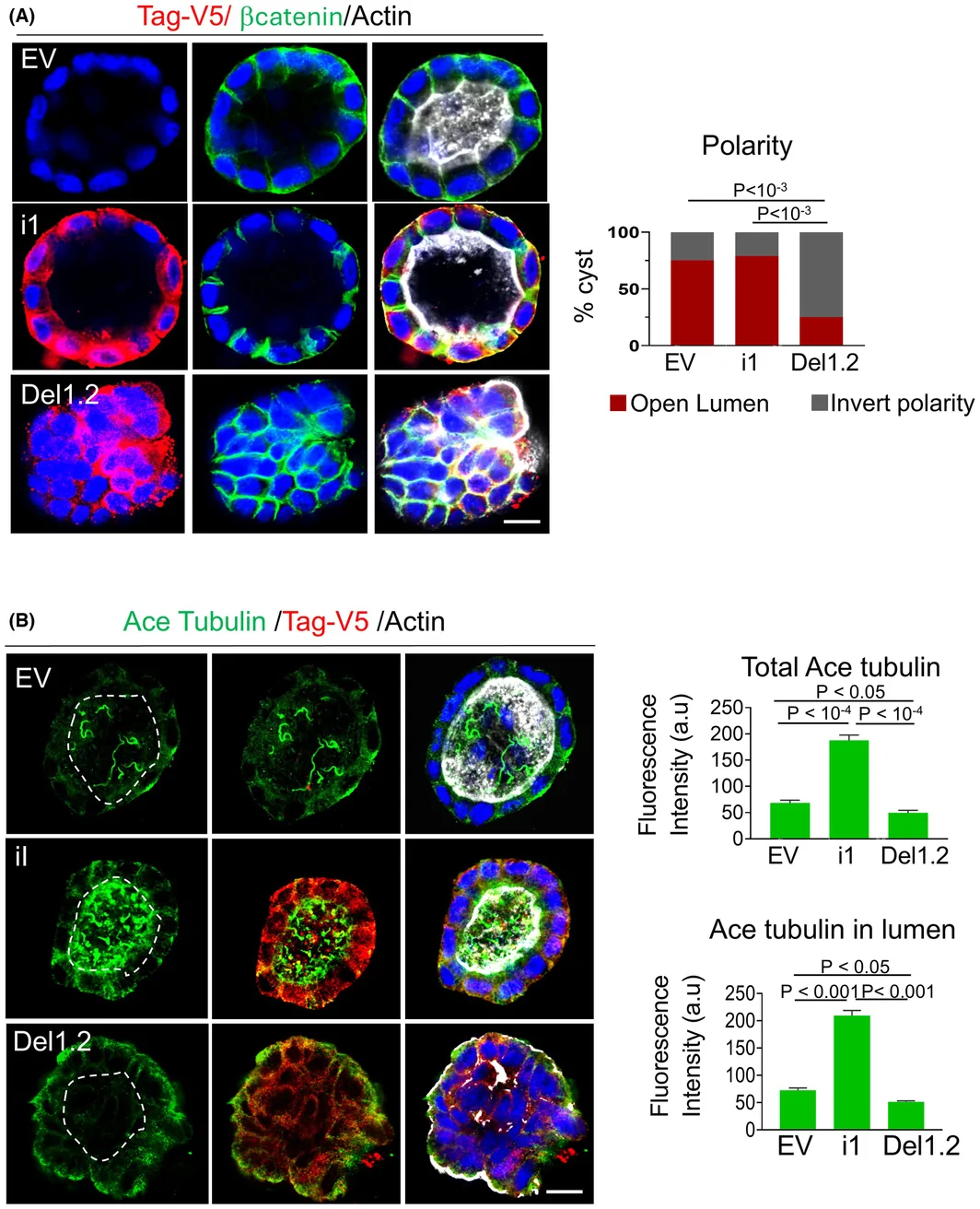
Septin 9 and its PB domains regulate ciliogenesis and epithelial polarity. (A) MDCK EV (Empty vector), i1, and septin 9_del1.2 cysts were stained for β‐catenin (green), V5‐tag (green), actin (gray), and nuclei (blue). Representative mid‐cyst sections and quantification of polarized versus nonpolarized (30 cysts/conditions, *n* = 3) Data are shown as mean ± SEM. Statistical analysis was performed using Student's *t*‐test (*P* < 0.05). Scale bar: 10 μm. (B) Cysts stained for acetylated α‐tubulin (Ace‐tubulin, green), V5‐tag (red), actin (gray), and nuclei. (blue). Luminal area (dashed circle) and acetylated α‐tubulin intensity were quantified. (15 cysts per condition, *n* = 3). Data are mean ± SEM. Statistical significance was assessed using Student's *t*‐test (*P* < 0.05).

### Septin 9 PB domains prevent HDAC6‐mediated microtubule deacetylation

Tubulin acetylation is negatively regulated by the tubulin deacetylase HDAC6 [33]. We hypothesized that deletion of septin 9 PB domains reduces α‐tubulin acetylation through aberrant activation of HDAC6 (Fig. 5A). Consistent with this hypothesis, immunoblot analysis revealed that expression of septin 9_del1,2 decreased acetylated tubulin levels while increasing HDAC6 expression (Fig. 5B). In 3D culture, HDAC6 was enriched apically in control (septin 9_EV) cysts but became strongly upregulated and mislocalized to the cell periphery in septin 9_del1.2 cysts (Fig. 5C). To directly assess the role of HDAC6, septin 9_del1,2 cysts were treated with the selective HDAC6 inhibitor tubacin. Remarkably, tubacin restored epithelial polarity, yielding cysts with large open lumens and luminal cilia (Fig. 5D). Immunoblotting confirmed a marked increase in acetylated tubulin levels and a decrease in HDAC6 expression following treatment (Fig. 5E). These findings demonstrate that hyperactivation of HDAC6 drives the microtubule deacetylation and polarity defects caused by loss of septin 9 PB domains.

**Fig. 5:**
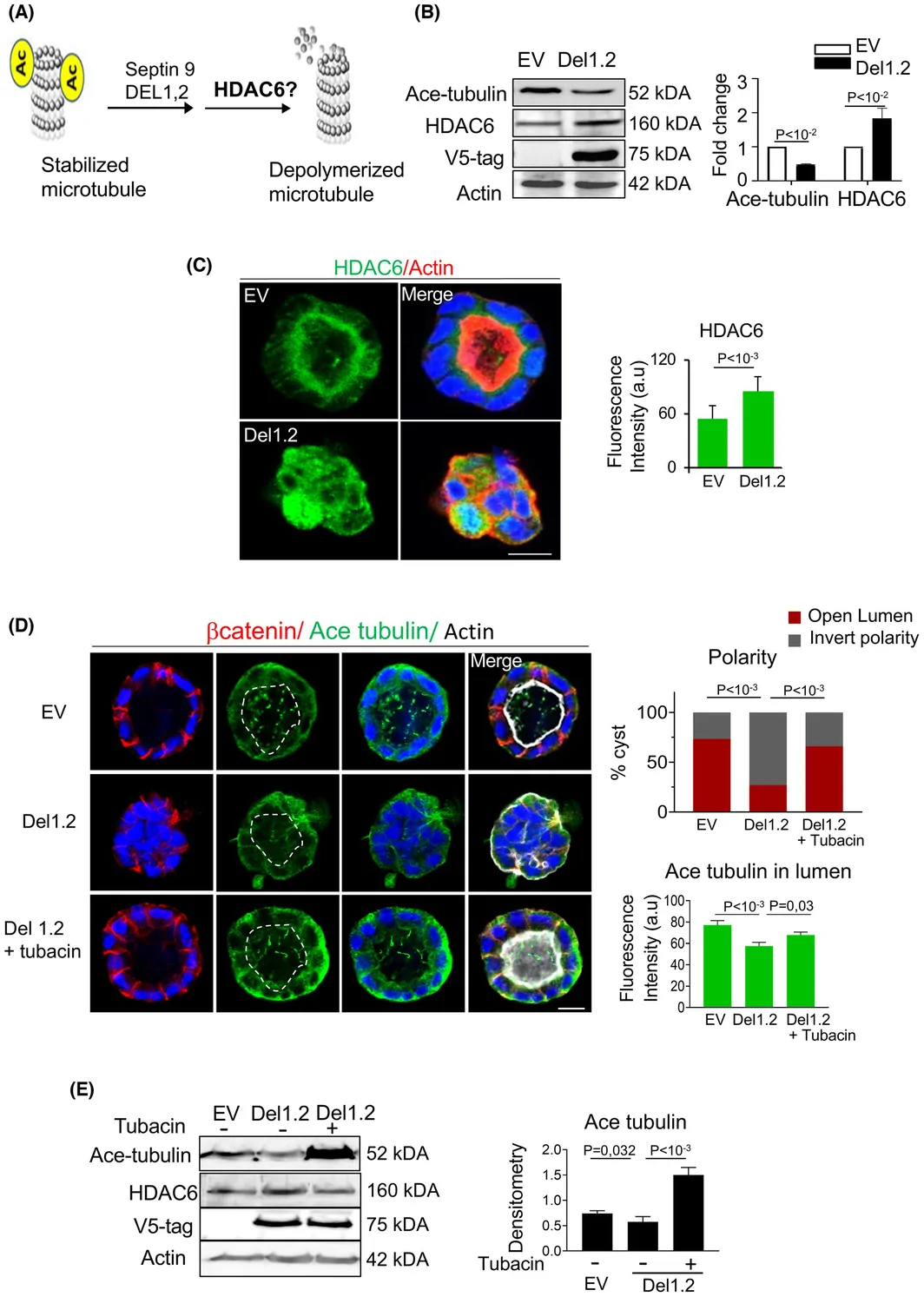
PB‐domain loss activates HDAC6‐dependent microtubule deacetylation. (A) Schematic of HDAC6‐mediated regulation of microtubule stability. (B) Immunoblot analysis of V5‐Septin 9, HDAC6, and acetylated α‐tubulin (Ace ‐tubulin) in MDCK EV (Empty vector) and septin 9_del1.2 cells. Data are representative of three independent experiments (*P* < 0.05). Actin was used as a loading control. (C) Cysts stained for HDAC6 (green), actin (red), and nuclei (blue). Scale bar: 10 μm Quantification of HDAC6 fluorescence (*n* > 10) Data are shown as mean ± SEM. Statistical analysis was performed using Student's *t*‐test (*P* < 0.05). (D) MDCK EV and Septin 9_del1.2 cysts treated with 1 μm tubacin were stained for acetylated α‐tubulin (green), β‐catenin (red), Actin (gray) and nuclei (blue). Scale bar: 10 μm. Polarity was quantified as the percentage of cysts showing open lumen versus inverted polarity (red: open lumen; gray: inverted polarity). Acetylated α‐tubulin intensity in the luminal area (dashed circle) was quantified (30 cysts per condition, three independent experiments). Data are shown as mean ± SEM. Statistical analysis was performed using Student's *t*‐test (*P* < 0.05). (E) Immunoblot analysis of cells treated with 2 μm tubacin showing levels of acetylated α‐tubulin, HDAC6, and V5‐Septin 9. Actin was used as a loading control [Experiments were performed independently three times (*n* = 3)]. Data are shown as mean ± SEM. Statistical analysis was performed using Student's *t*‐test (*P* < 0.05).

### TGF‐β signaling mediates HDAC6 activation and polarity defects

We previously reported that deletion of septin 9 PB domains activates TGF‐β signaling, induces EMT and inverted polarity [26]. Because HDAC6 is a key effector of TGF‐β‐induced EMT [34, 35] and TGF‐β signaling promotes HDAC6‐dependent microtubule deacetylation [36] (Fig. 6A), we investigated whether TGF‐β signaling contributes to the septin 9_del1.2 phenotype. Treatment of septin 9_del1.2 cells with the TGF‐β receptor I inhibitor SB431542 restored epithelial polarity as previously reported [26] with apical relocalization of both acetylated tubulin and HDAC6 (Fig. 6B–D). Notably, SB431542 treatment also repositioned the centrosome asymmetrically in association with acetylated microtubules (Fig. 6E–G). Immunoblotting and qRT‐PCR analyses confirmed reduced HDAC6 expression and increased acetylated tubulin levels (Fig. 6H; Fig. S4A,B). Sirtuin 2 (SIRT2), another tubulin deacetylase [37, 38], exhibited similar TGF‐β‐dependent regulation (Fig. S4A). In addition, expression of Human Enhancer of Filamentation 1 (HEF1/NEDD9), a cilia‐disassembly scaffold [6], was elevated in septin 9_del1.2 cysts and reduced upon TGF‐β inhibition (Fig. S4B). Collectively, these results demonstrate that septin 9 PB domains preserve epithelial polarity by restraining TGF‐β/HDAC6‐mediated microtubule deacetylation and EMT.

**Fig. 6:**
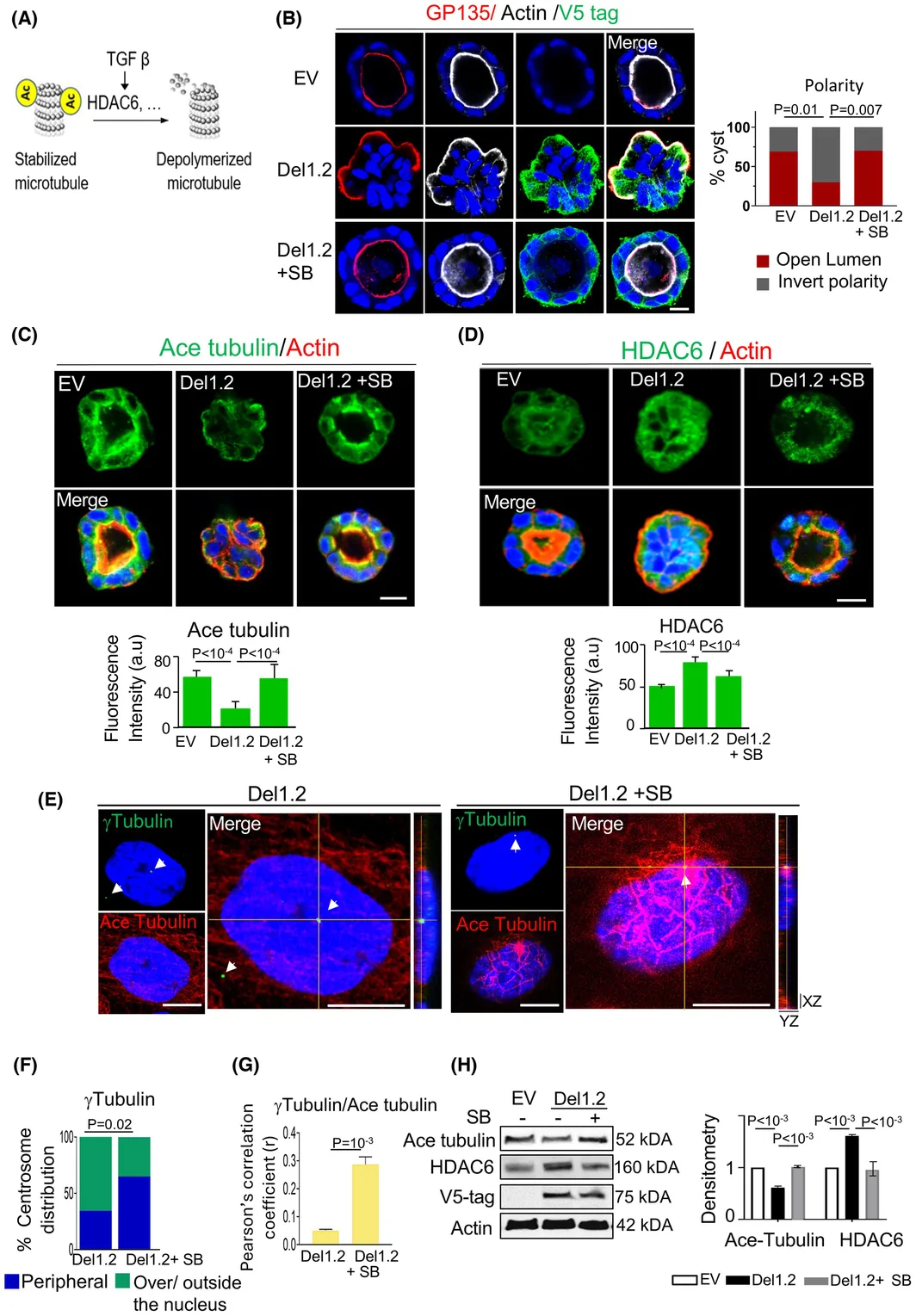
TGF‐β signaling mediates septin 9‐dependent microtubule and centrosome asymmetry. (A) Schematic linking TGF‐β signaling to HDAC6‐mediated microtubule regulation. (B) MDCK EV (empty vector) and Septin 9_del1.2 cysts grown in 3D culture are shown. Septin 9_del1.2 cysts were treated with SB431542 (2 μm). Cells were stained for GP135 (red), actin (gray), V5‐tag (green), and nuclei (blue). Scale bar: 10 μm. Polarity was quantified as the percentage of cysts showing open lumen versus inverted polarity (red: open lumen; gray: inverted polarity). Quantification is shown from 30 cysts per condition across 3 independent experiments. Data are presented as mean ± SEM. Statistical analysis was performed using Student's *t*‐test (*P* < 0.05). (C) MDCK EV and Septin 9_del1.2 cysts were treated with 2 μm SB431542 and stained for acetylated α‐tubulin (Ace‐tubulin, green), actin (red), and nuclei (blue). Scale bar: 10 μm. Quantification of polarity and acetylated α‐tubulin levels (Counts of 30 cysts were obtained from three independent experiments). Data are shown as mean ± SEM. Statistical analysis was performed using Student's t‐test (*P* < 0.05). (D) SB431542‐treated cysts stained for HDAC6 (green), actin (red), and nuclei, with fluorescence quantification (*n* > 10). Scale bar: 10 μm. Data are shown as mean ± SEM. Statistical analysis was performed using Student's *t*‐test (*P* < 0.05). (E) Cells stained for γ‐tubulin (green), acetylated α‐tubulin (red), and nuclei (blue) after SB431542 treatment at 2 μm. White arrowheads indicate centrosomes. XZ projections show centrosome positioning. Scale bar: 10 μm. (F) Quantification of centrosome distribution patterns. Data are expressed as percentages from 40 cells per condition in independent experiments (*n* = 3). Data are shown as mean ± SEM. Statistical analysis was performed using Student's *t*‐test (*P* < 0.05). (G) Colocalization coefficients were calculated under the indicated conditions using Pearson's correlation coefficient. Data are shown as mean ± SEM. Statistical analysis was performed using Student's *t*‐test (*P* < 0.05). (H) Immunoblot of acetylated α‐tubulin and HDAC6 after SB431542 treatment at 2 μm (three experiments; *P* < 0.01). Actin was used as a loading control. Data are shown as mean ± SEM. Statistical analysis was performed using Student's *t*‐test (*P* < 0.05).

## Discussion

Cell polarity is fundamental to epithelial tissue organization, ciliogenesis, and directional signaling and depends on the coordinated regulation of cytoskeletal architecture, membrane trafficking, and organelle positioning. In this study, we identify septin 9 and its two PB domains as critical regulators of centrosome positioning, asymmetric microtubule acetylation, and apico‐basal polarity, acting through HDAC6 and TGF‐β signaling. Septins are increasingly recognized as cytoskeletal scaffolds that organize actin and microtubules into higher‐order structures to control intracellular trafficking and organelle positioning [21, 39].

Septin 9, a component of the canonical septin 2‐6‐7‐9 octameric complex, has been implicated in Golgi organization, ciliogenesis, and epithelial polarity [25, 26]. Our findings extend these roles by demonstrating that septin 9 filaments converge at the centrosomal region and colocalize with acetylated microtubules, revealing a structural and regulatory role in organizing stabilized, post‐translationally modified microtubule populations. Loss of septin 9 or deletion of its PB domains disrupted centrosome positioning and resulted in a symmetric, perinuclear distribution of acetylated tubulin, supporting a role for septins in enforcing cytoskeletal asymmetry. In 3D MDCK cysts, PB‐domain deletion impaired ciliogenesis and reduced acetylated tubulin levels, consistent with the established role of acetylated microtubules in primary cilium stability and function [32]. Together, these results indicate that septin 9‐dependent organization of acetylated microtubules is required to spatially couple centrosome positioning with apical ciliogenesis and lumen formation.

Mechanistically, reduced microtubule acetylation in septin 9‐depleted or PB‐domain‐deficient cells was associated with increased expression and mislocalization of HDAC6, a major cytoplasmic tubulin deacetylase [33] and a key effector of TGF‐β‐induced epithelial‐to‐mesenchymal transition [34, 36]. Inhibition of TGF‐β signaling restored acetylated tubulin levels and epithelial polarity, while selective HDAC6 inhibition with tubacin rescued microtubule acetylation, centrosome positioning, and apico‐basal organization. These findings establish hyperactive HDAC6 as a central mediator of the polarity defects caused by loss of septin 9 PB domains.

Previous work showed that septin 7 depletion in neurons increases microtubule acetylation by impairing HDAC6 scaffolding, thereby fine‐tuning microtubule stability during neuritogenesis [40]. Importantly, that study emphasized cell‐type specificity, as epithelial cells exhibited distinct regulatory behaviors. In our epithelial system, deletion of septin 9 PB domains led to reduced septin 7 expression (Fig. S3), suggesting destabilization of the septin complex. We propose that loss of septin‐complex integrity in septin 9_del1.2 cells indirectly promotes HDAC6 upregulation and mislocalization, resulting in microtubule deacetylation, centrosome mispositioning, and polarity defects. Although we did not directly assess HDAC6‐septin 7 interactions, the diffuse peripheral distribution of septin 7 is consistent with impaired complex assembly. These observations underscore that, while septin‐HDAC6 regulatory mechanisms differ between neuronal and epithelial contexts, both rely on septin‐complex integrity to control microtubule acetylation and cytoskeletal organization.

Beyond HDAC6, the reduction of SIRT2 and HEF1 expression upon TGF‐β inhibition suggests that septin 9 counteracts multiple TGF‐β‐dependent pathways that destabilize microtubules and impair ciliogenesis [6, 37, 41]. We propose that septin 9 acts as a cytoskeletal integrator that enforces asymmetric microtubule acetylation to support centrosome and Golgi positioning, thereby enabling polarized vesicle trafficking and cilia formation, key processes underlying epithelial morphogenesis [11, 28, 42]. Disruption of this regulatory axis leads to inverted polarity, EMT‐like features, and enhanced HDAC6‐mediated deacetylation, consistent with links between septin dysfunction, epithelial barrier defects, and invasive phenotypes [26, 42]. In summary, our findings identify the PB domains of septin 9 as essential regulators of septin‐complex integrity, HDAC6 activity, and asymmetric microtubule acetylation. By coupling centrosome positioning to stabilized microtubule networks and ciliogenesis, septin 9 enforces epithelial polarity. These results provide a mechanistic framework connecting septins to cytoskeletal asymmetry and epithelial morphogenesis, with potential implications for understanding polarity defects in development and disease.

## Author contributions

TC: conceptualization, methodology, writing, formal analysis, performed the experiments, prepared the digital images. MO: methodology, writing, formal analysis, performed the experiments, and prepared the digital images. NB: performed the experiments, analyzed the data, and prepared the digital images. DS: supervision; funding acquisition. AGD: conceptualization; supervision; funding acquisition, investigation, and writing. All authors have read and agreed to the published version of the manuscript.

## Supporting information


**Fig S1.** PB‐domain deletion disrupts Golgi/centrosome and microtubule asymmetry in HeLa cells.
**Fig. S2.** Effect of nocodazole on microtubules in MDCK cells.
**Fig. S3.** PB‐domain deletion reduces septin 7 levels and alters its localization.
**Fig. S4.** PB‐domain deletion affects SIRT2 and HEF1 expression.
**Table S1.** Primer sequences.

## Data Availability

The datasets supporting the current study have not been deposited in a public repository but are available upon request from the corresponding author.
