# Supplementary material for: Septin 9 PB domains coordinate centrosome positioning and microtubule acetylation to control epithelial polarity
Source: FEBS Lett. 2026 May 26;600(20):3073–88. doi: 10.1002/1873-3468.70363 (PMC13703879; doi:10.1002/1873-3468.70363)
Supplement: Supplementary file 1 [file FEB2-600-3073-s001.pdf]

Primer name	Forward sequence	Reverse sequence
**HDAC6**	AAGAAGACCTAATCGTGGGACT	GCTGTGAACCAACATCAGCTC
**SIRT2**	CTGCGGAACTTATTCTCCCAGAC	CCACCAAACAGATGACTCTGCG
**GAPDH**	AGC CAC ATC GCT CAG ACA C	GCC CAA TAC GAC CAA ATC C

Supplementary Table S1.

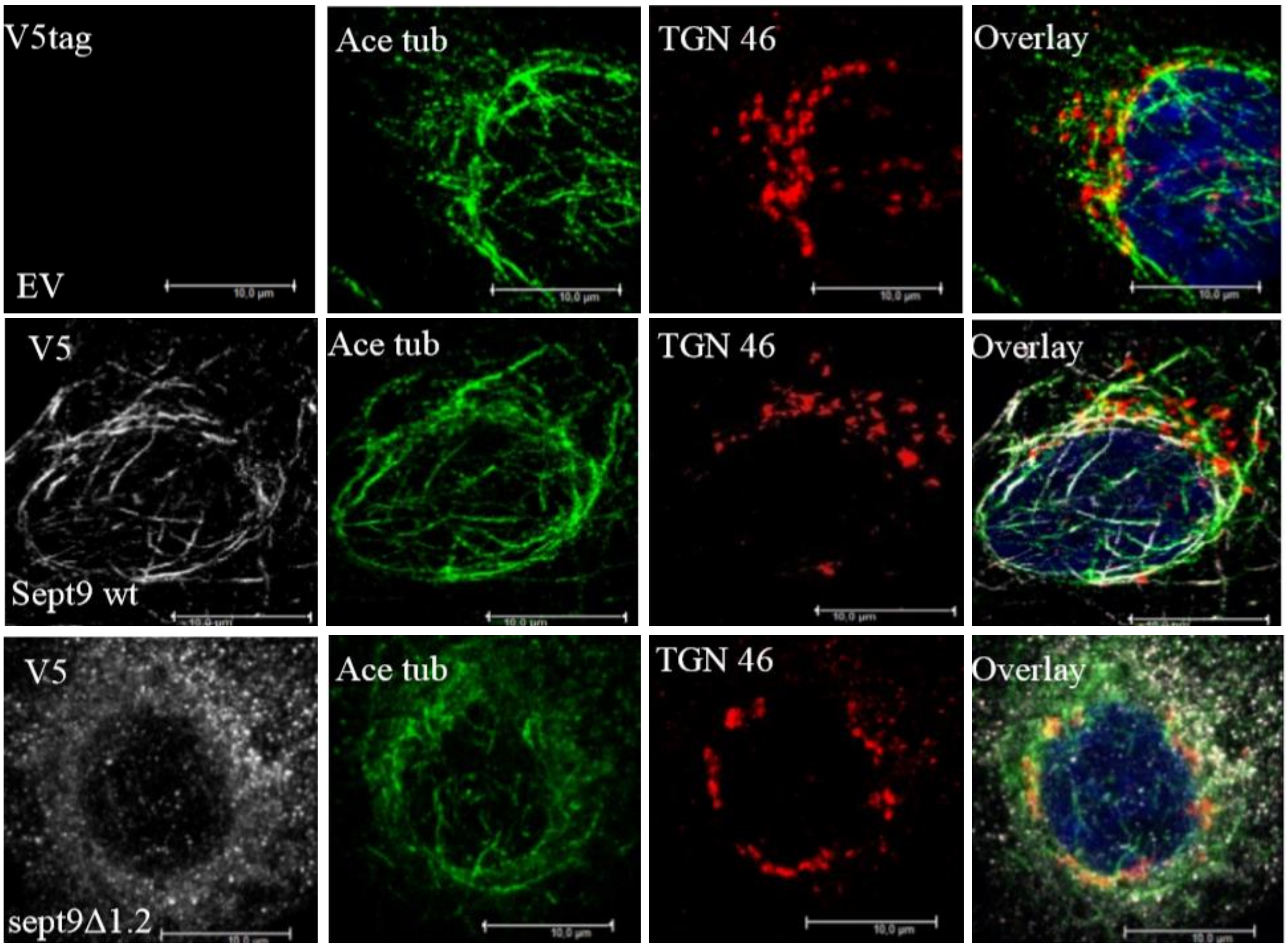

Supplementary Fig. S1. PB-domain deletion disrupts Golgi/centrosome and microtubule asymmetry in HeLa cells.

Hela cells transfected with EV (empty vector), WT septin 9_del1.2 were stained for V5 (gray), TGN46 (red), acetylated α-tubulin (Ace tub, green) and nuclei (blue). Scale bar: 10 µm.

Supplementary Fig.S2

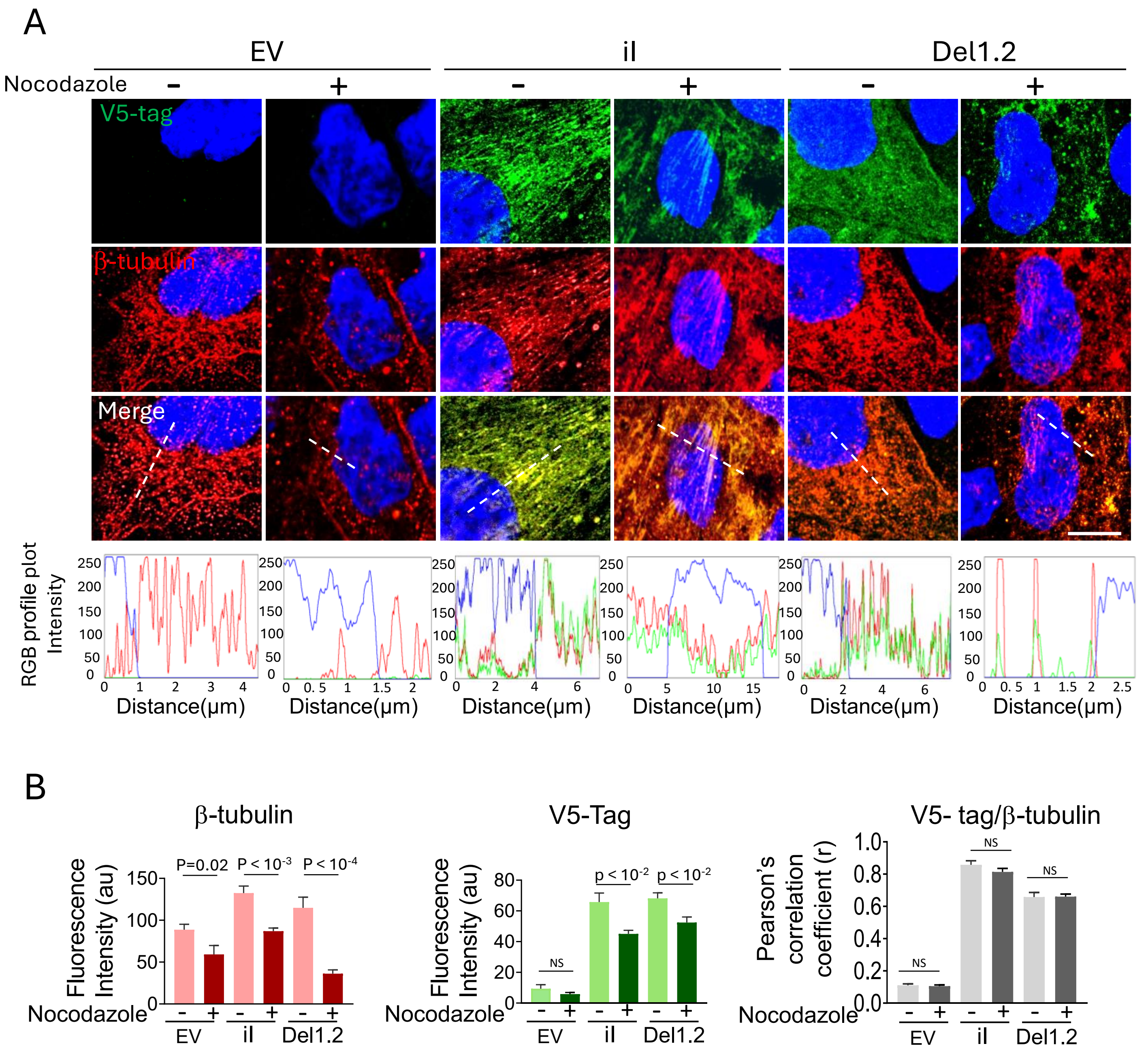

Supplementary Fig.S2. Effect of nocodazole on microtubules in MDCK cells. (A) MDCK cells expressing EV(empty vector), septin 9 (i1), or septin 9_del1.2 were cultured in 2D, treated or not with 33 nM nocodazole for 2 h, and stained for V5-tag (green), β-tubulin (red), and nuclei (blue). Below RGD profiles were obtained from merged images along the dashed line using the Fiji RGD Profile plugin. Scale bar: 10 μm. Data are shown as mean ± SEM. Statistical analysis was performed using Student's t-test ($p < 0.05$). (B) Quantification of β-tubulin and V5-tag fluorescence levels (counts of 30 cells from three independent experiments) and colocalization analysis (counts of 10 cells from three independent experiments). Data are shown as mean ± SEM. Statistical analysis was performed using Student's t-test ($p < 0.05$).

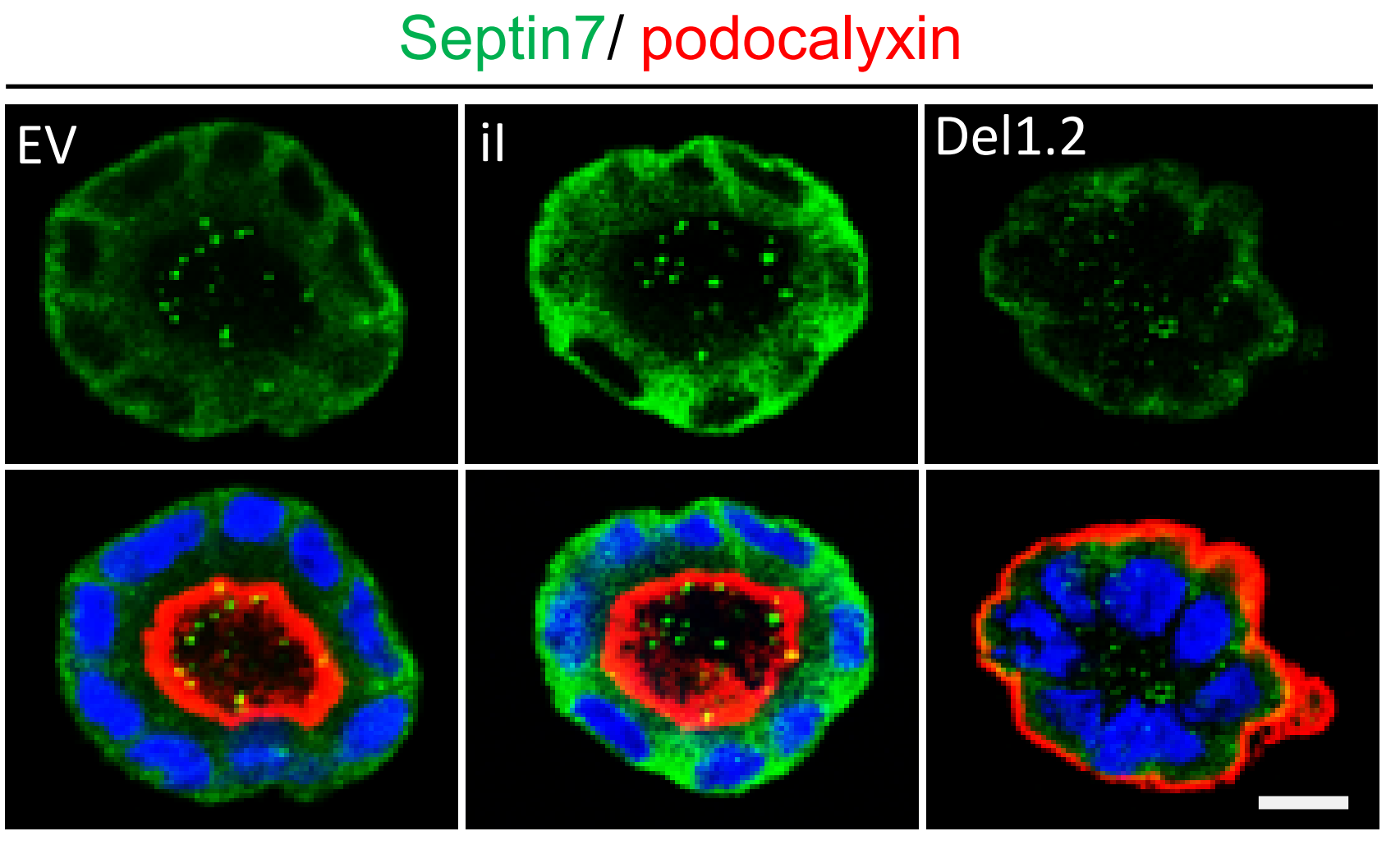

Supplementary Fig. S3. PB-domain deletion reduces septin 7 levels and alters its localization.

3D cultures of MDCK cells expressing EV (empty vector), septin 9 (i1), or septin 9_del1.2 were analyzed. Cysts were stained for septin 7 (green), podocalyxin (red), and nuclei (blue). Scale bar: 10 µm.

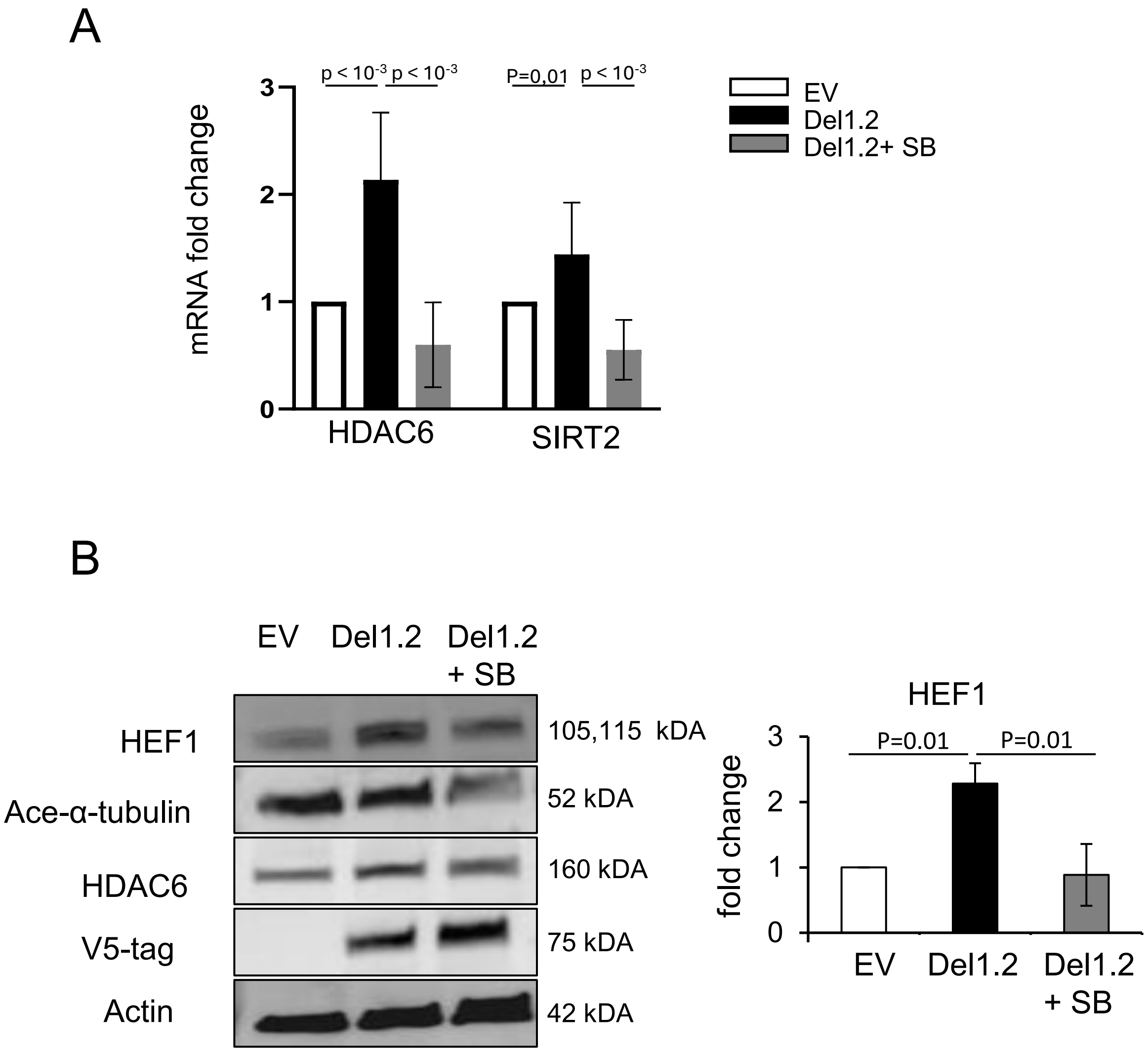

Supplementary Fig. S4. PB-domain deletion affects SIRT2 and HEF1 expression.

(A) RT-qPCR of HDAC6 and SIRT2 mRNA in EV and septin 9_del1.2 cells treated with SB431542. Data are shown as mean ± SEM. Statistical analysis was performed using Student's t-test ($p < 0.05$). **(B)** Immunoblot of HEF1, HDAC6, and V5–septin 9 in EV and septin 9_del1.2 cells treated with SB431542 at 2µM (n = 3). Data are shown as mean ± SEM. Statistical analysis was performed using Student's t-test ($p < 0.05$). n.s. indicates not statistically significant. Actin was used as a loading control.
